# Endoscopic papillary large balloon dilatation‐assisted retrieval of a proximally migrated double pigtail stent from the bile duct: A case report

**DOI:** 10.1002/deo2.272

**Published:** 2023-07-10

**Authors:** Hidehiro Kamezaki, Hiroshi Yoshikawa, Terunao Iwanaga, Mamoru Tokunaga, Takahiro Maeda, Junichi Senoo, Hiroshi Ohyama, Naoya Kato

**Affiliations:** ^1^ Department of Gastroenterology Eastern Chiba Medical Center Chiba Japan; ^2^ Department of General Medicine Eastern Chiba Medical Center Chiba Japan; ^3^ Department of Gastroenterology Graduate School of Medicine Chiba University Chiba Japan

**Keywords:** choledocholithiasis, endoscopic retrograde cholangiopancreatography, endoscopic sphincterotomy, stent migration, stents

## Abstract

Double‐pigtail stents are commonly used for drainage in cholangitis to prevent stent migration. We report a case in which a double pigtail stent had migrated proximally into the bile duct and was successfully retrieved after endoscopic papillary large balloon dilatation (EPLBD). An 86‐year‐old man underwent endoscopic papillary sphincterotomy for cholangitis due to common bile duct stones and had a double‐pigtail stent placed in the common bile duct. The patient presented a week later for endoscopic biliary stone removal, but the stent had migrated proximally and could not be visualized during the endoscopy. Endoscopic papillary large balloon dilatation was performed to dilate the papilla to a diameter of 12 mm, following which the stent was grasped and removed. The biliary calculi were subsequently extracted, and the procedure was completed without any complications. This case highlights the potential usefulness of endoscopic papillary large balloon dilatation for retrieving a double‐pigtail stent that has migrated to the bile duct.

## INTRODUCTION

Stent migration is a known complication of endoscopic retrograde cholangiopancreatography with stent placement, and previous studies have reported varying rates of stent migration.^1^ Double‐pigtail stents are commonly used for drainage in cholangitis to prevent migration, but despite their design, migration of the stent has been reported in some studies, often in the distal direction.^2^, ^3^ In this case report, we describe the successful use of endoscopic papillary large balloon dilatation (EPLBD) to help retrieve a double‐pigtail stent that had migrated proximally into the bile duct.

## CASE PRESENTATION

An 86‐year‐old man presented to our hospital for endoscopic biliary stone removal. The patient had a history of postoperative gastric cancer following distal gastrectomy and Billroth I reconstruction, as well as hypertension, and had been taking oral medications, such as nifedipine, amlodipine, and esomeprazole.

**FIGURE 1:**
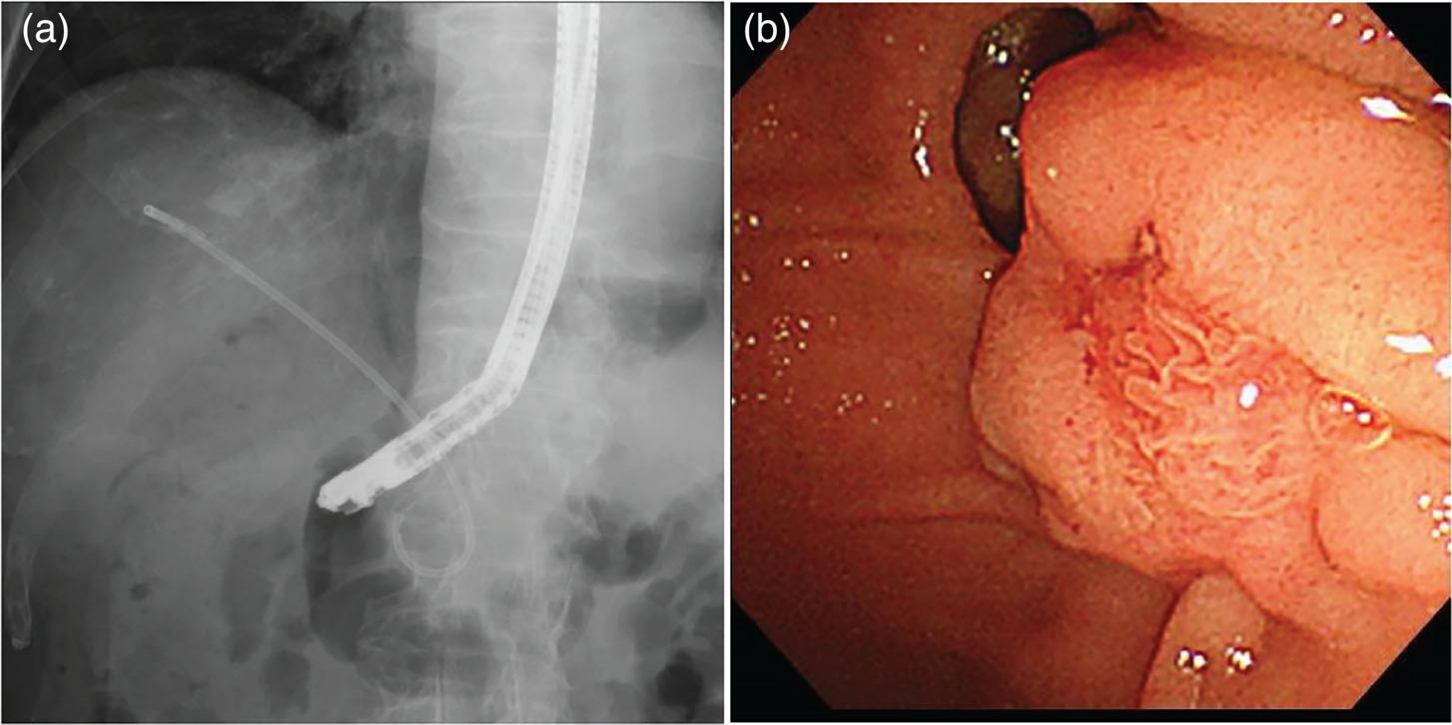
One day after admission, an abdominal X‐ray showed that the patient had a double pigtail stent placed in the bile duct. During papilla observation, esophagogastroduodenoscopy could not locate the bile duct stent suggesting that the stent had migrated into the bile duct.

**FIGURE 2:**
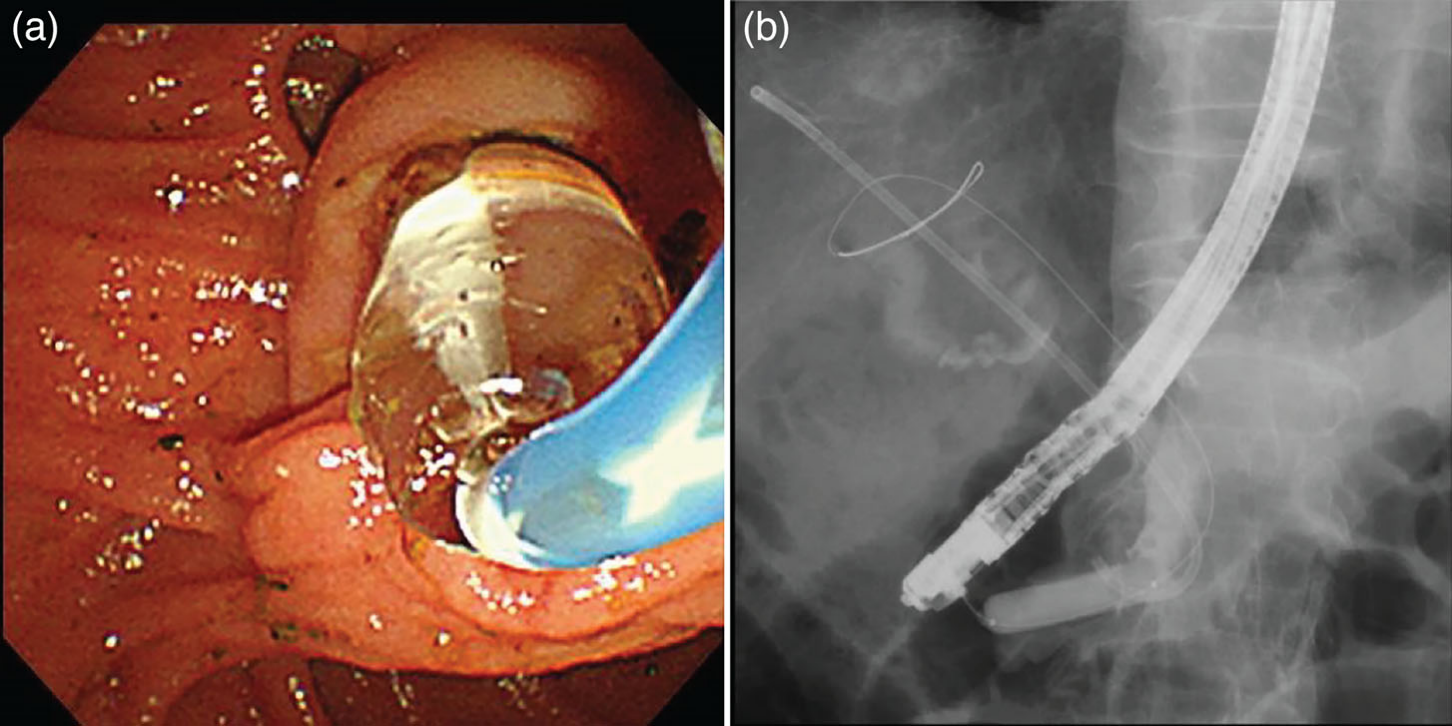
Endoscopic papillary large balloon dilatation (EPLBD) was performed, which expanded the papilla to a diameter of 12 mm.

**FIGURE 3:**
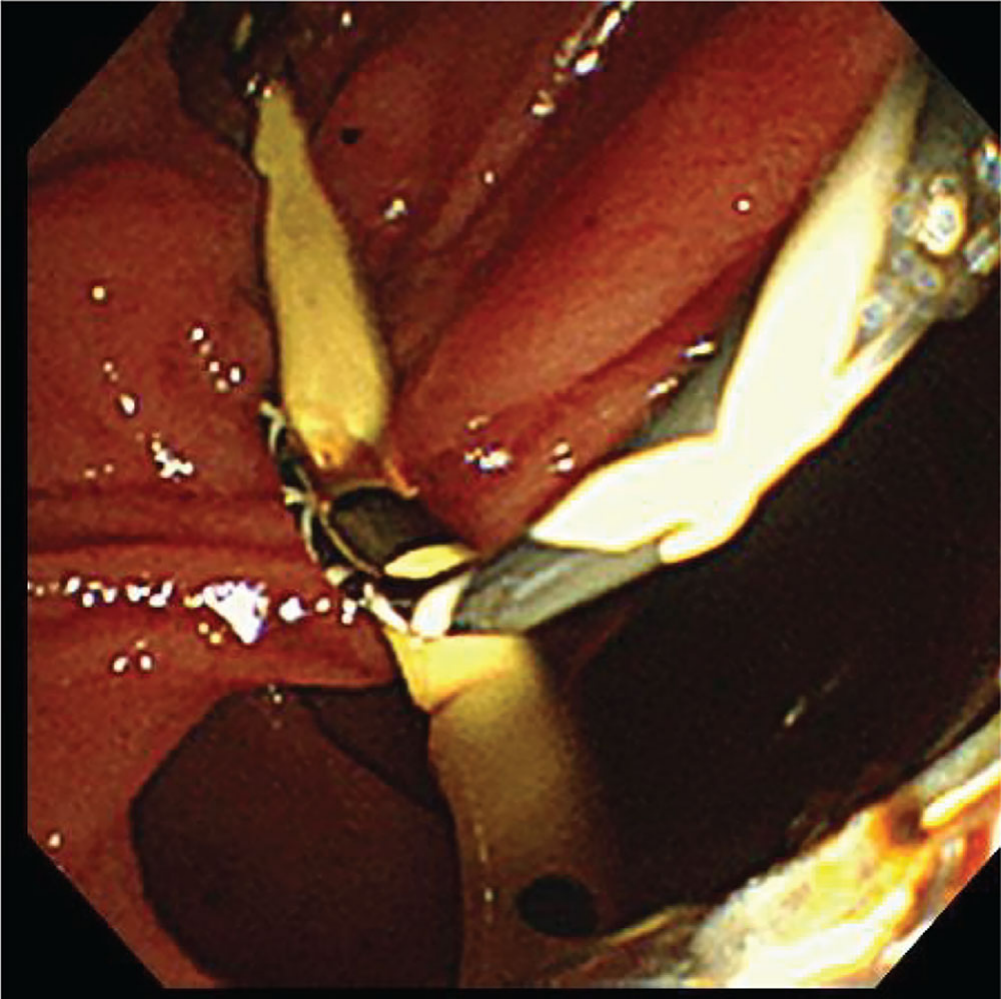
Following endoscopic papillary large balloon dilatation, grasping forceps were inserted into the bile duct, and the stent was successfully grasped and retrieved through the papilla.

Six days prior to the presentation, the patient underwent endoscopic papillary sphincterotomy at a different hospital. A double‐pigtail stent (details not available) was placed in the bile duct for cholangitis due to common bile duct stones. The patient was discharged with the advice of follow‐up for biliary stone removal once the cholangitis subsided. The patient was admitted to our hospital for endoscopic biliary stone removal. Endoscopic retrograde cholangiopancreatography was performed the next day. Although the abdominal radiograph showed that the patient had a double‐pigtail stent with a loop diameter of approximately 24 mm (Figure 1), the stent could not be visualized during endoscopic observation of the papilla, indicating that the stent had migrated into the bile duct where its looped end was likely caught within the papilla (Figure 1). Stent retrieval was attempted using various balloon and basket catheters; however, it proved challenging and was unsuccessful owing to the narrow papilla. The presence of papillary stenosis and the limited range of motion of the retrieval devices, due to the procedure being performed under X‐ray fluoroscopy, hindered the insertion of forceps into the bile duct to firmly grasp the distal end. Therefore, endoscopic papillary large balloon dilatation (EPLBD) was performed, wherein the papilla was dilated to a diameter of 12 mm (Figure 2). Grasping forceps were inserted into the bile duct, and the stent was successfully grasped and retrieved through the papilla (Figure 3). EPLBD improved the maneuverability of grasping forceps within the bile duct, ensuring a reliable grasp of the distal end of the stent, and eliminating resistance during extraction. Biliary calculi were also removed, and the treatment was completed without complications. The patient resumed eating the next day, intravenous fluids were stopped the same day, and the patient was discharged on a subsequent day.

## DISCUSSION

Stent migration is a known complication of endoscopic retrograde cholangiopancreatography with stent placement, with varying rates reported in previous studies. According to a review by Dumonceau et al., which was used to develop the European Society of Gastrointestinal Endoscopy clinical guidelines, the migration rate for plastic stents and partially covered self‐expandable metal stents is around 5%. In comparison, uncovered self‐expandable metal stents have a migration rate of 1%, and fully covered self‐expandable metal stents have a rate of 20%.^1^ Stent migration can occur either proximally or distally within the bile duct. Risk factors for proximal migration include malignant strictures, a larger bile duct diameter, distally located strictures, and stent‐related factors such as using larger‐diameter, short, or straight stents.^4^

Double‐pigtail stents have been developed to prevent or reduce stent migration and are used alone or to anchor other stents.^2^ Temporary biliary stenting using a double‐pigtail stent has been a safe and feasible method for treating difficult and large common bile duct stones refractory to conventional methods.^3^, ^5^ It can serve as a bridge therapy to secondary interventions. However, few cases of migration of double‐pigtail stents have been reported, typically in the distal direction, in elderly patients,^5^, ^6^ and prolonged use.^3^

**FIGURE 4:**
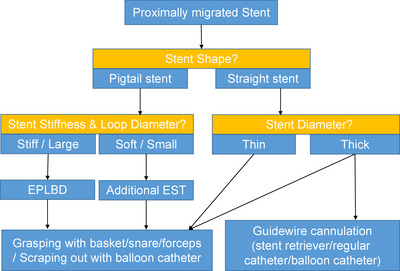
Flowchart depicting treatment choices for a proximally migrated biliary stent. Stent shape, size, and stiffness should be considered to decide the treatment approach, such as using endoscopic papillary large balloon dilatation (EPLBD) with or without additional endoscopic sphincterotomy (EST) or guidewire cannulation, before stent retrieval using baskets, snares, forceps, or catheters.

While most cases of distal stent migration lead to the spontaneous passage, proximal migration of stents into the bile duct and pancreatic duct requires endoscopic extraction.^7^, ^8^ Different devices such as basket catheters, balloon catheters, grasping forceps, guidewires, and lithotripsy tools can be used to retrieve the migrated stent. However, the appropriate treatment method should be chosen based on the individual case, depending on stent shape, stiffness, and size (Figure 4). In this case, retrieval of the pigtail stent with balloon and basket catheters was challenging due to the stent being caught in the narrow papilla. Considering the narrow papilla and the large, possibly stiff loop of the double pigtail stent, we decided to use EPLBD instead of endoscopic sphincterotomy to expand the duodenal papilla and retrieve the stent using grasping forceps. To the best of our knowledge, this is the first report of a case of a migrated double‐pigtail stent that was treated using an EPLBD, expanding the papilla to allow stent removal. A PubMed search for “migration,” “stent,” and “pigtail” revealed four cases, but there were no reports using EPLBD. Ozair et al. reported a case in which a double‐pigtail stent had undergone transhepatic migration through the right hepatic duct and protruded into the peritoneal cavity. Retrieval was performed at the time of cholecystectomy.^9^ Kawaguchi et al. reported three cases of proximally migrated biliary tube stents removed using snares, baskets, forceps, or retrievers without resorting to EPLBD.^10^

In our case, EPLBD proved to be a successful method for treating a narrow papilla and helping retrieve a migrated double‐pigtail stent from the bile duct when conventional methods such as balloon catheters and basket catheters proved difficult. This experience suggests that EPLBD may be a useful step in the treatment of proximally migrated double‐pigtail stents in challenging cases, particularly in scenarios with a narrow papilla and a stiff, large stent loop.

## CONFLICT OF INTEREST STATEMENT

None.
